# A semiquantitative study of the optimal whole-body imaging time after ^131^I therapy for differentiated thyroid cancer

**DOI:** 10.3389/fendo.2022.955387

**Published:** 2022-08-25

**Authors:** Shuang Liu, Rui Zuo, Tianyu Yang, Hua Pang, Zhengjie Wang

**Affiliations:** Department of Nuclear Medicine, The First Affiliated Hospital of Chongqing Medical University, Chongqing, China

**Keywords:** differentiated thyroid carcinoma, post-therapy ^131^I whole-body scan, semiquantitative study, visual analysis, optimal imaging time

## Abstract

**Objective:**

We compared the efficacy of post-therapy whole-body scintigraphy (Tx-WBS) in terms of detecting lesions in patients with differentiated thyroid cancer (DTC) on days 3, 7, and 10 after ^131^I treatment, and we determined the optimal imaging time.

**Methods:**

Clinical data from 161 DTC patients treated with ^131^I were collected. All patients underwent day 3 imaging, but only 98 patients underwent day 3 and day 7 imaging, and 63 patients underwent day 3 and day 10 imaging at the same time. And the thyroid bed uptake was visually graded. The radioactivity ratios of the thyroid bed, neck lymph nodes, lungs, and liver (to the background) were calculated to allow a semiquantitative analysis.

**Results:**

Visual analysis showed that delayed imaging revealed more lymph node and lung radioactivity, early imaging showed more residual thyroid tissue, and significant differences in uptake were apparent at days 3, 7, and 10 (*P* < 0.001). Semiquantitative analysis revealed significant differences in the target-to-background ratios of the residual thyroid bed, lungs, and liver at days 3, 7, and 10. On these days, the imaging sensitivities in terms of detecting metastatic lymph nodes were 29.58%, 39.02%, and 19.35%, and the specificities were 75.56%, 75.86%, and 75% (*P* = 0.465, 0.154, and 0.763, respectively). In terms of lung metastasis detection, the sensitivities were 29.58%, 38.46%, and 13.33% respectively, and the specificities were 98.33%, 100%, and 95.83% (*P* < 0.001, < 0.001, and *P*=0.238).

**Conclusion:**

More residual thyroid tissue can be detected by imaging on day 3; imaging on day 7 more effectively detects lung metastases than does imaging on day 3 or 10.

## Introduction

A thyroid carcinoma is a malignant tumor originating from the thyroid follicular epithelium or parafollicular epithelial cells. In 2022, thyroid cancer was the seventh most common female tumor, with an incidence of 3% (1), and one of the most common malignant tumors of the head-and-neck endocrine system. Differentiated thyroid cancer (DTC) accounts for more than 95% of all thyroid carcinomas (2). The overall mortality of DTC patients is increasing; some cancer subtypes are prone to extrathyroidal and vascular invasion, high-level recurrence, and metastasis. The prognosis is thus poor (3). Early detection of recurrence and metastasis is important. The 2015 American Thyroid Association (ATA) Management Guidelines for Adult Patients with Thyroid Nodules and Differentiated Thyroid Cancer (4) state that radionuclide ^131^I therapy can be performed after DTC surgery to remove residual thyroid tissue, reduce the risks of tumor recurrence and death, facilitate follow-up monitoring and timely clinical restaging, and guide follow-up treatment decisions. A post-therapy ^131^I whole-body scan (Tx-WBS) after ^131^I treatment evaluates whether residual thyroid tissue has been ablated and detects recurrent or metastatic lesions. The ATA guidelines and those of other societies suggest that Tx-WBS should be performed 2–10 days after ^131^I treatment (4–7). However, the optimal time remains unknown. Currently, DTC patients undergo multiple examinations; this increases the radiation dose, hospitalization time, and cost. Therefore, it is very necessary for DTC patients to find an optimal imaging time.

Qiu et al. (8) found that the detection capacity of Tx-WBS varied significantly over the 3 consecutive days after ^131^I treatment for metastatic thyroid cancer; ^131^I uptake increased linearly, suggesting that lesional detection capacity was related to imaging time. However, no study has compared lesional detection in a large patient sample over 2–10 days; no consensus on the optimal imaging time has emerged. Here, we compared the efficacies of imaging at different times; we provide a theoretical basis for DTC follow-up and treatment.

## Material and methods

### Study population

This was a retrospective review. A total of 161 patients with DTC treated with ^131^I in our department from December 2019 to March 2021 were enrolled; 94.4% had papillary cancer. Overall, 68 patients were male, 93 were female, and the mean age was 44.75 ± 14.43 (12–79) years. All stopped taking thyroxine and consumed a low-iodine diet for 4 weeks prior to therapy [to increase the level of thyroid stimulating hormone (TSH) to ≥ 30 µIU/mL]. All patients received oral ^131^I (100–200 mCi) after formulating personalized treatment plan according to the preoperative evaluation of thyroglobulin (Tg) and antithyroglobulin antibody (TgAb) levels, and neck ultrasound. Tg was measured using a chemiluminescence method (Beckman Coulter Co. Ltd., USA) (range 0–50.03 ng/mL) and thyroid function was analysed *via* radioimmunoassay (Beijing North Institute of Biotechnology Co. Ltd., China). The TSH range was 0.3–5.0 mIU/mL, the TgAb range was 0–4 IU/mL, and the anti-thyroid peroxidase antibody range was 0–9 IU/mL.

### ^131^I whole-body imaging

A total of 161 patients underwent whole-body imaging on the 3rd day after ^131^I treatment, of which 98 patients underwent both 3rd and 7th day imaging, and 63 patients underwent both 3rd and 10th day imaging using a Siemens Symbia T2 single-photon emission computed tomography/computed tomography (SPECT/CT) instrument equipped with a high-energy, parallel hole universal collimator (energy peak 364 keV, window width 20%, magnification 1.0, matrix 256 × 1,024, and scanning speed 15 cm/min). The early imaging was the whole-body imaging on the 3rd day after 131 iodine treatment, and the delayed imaging was the whole body imaging on the 7th and 10th days after 131 iodine treatment. The same two senior nuclear medicine specialists, who were unware of reconstruction protocol, independently and randomly evaluated all Whole-body images. ^131^I uptake by the oral and nasal cavities, gastrointestinal tract, and salivary glands were physiological; uptake by the residual thyroid and other foci was pathological. Thyroid bed uptake was visually graded: I = not visible; II = suspicious; or III = clearly visible. Circular regions of interest (ROIs) were manually drawn on the forehead, thyroid bed, lungs, and liver of frontal whole-body images and the target-to-background ratios (TBRs) calculated (the forehead served as the background). Recurrence or metastasis was examined *via* puncture biopsy, SPECT/CT imaging, cervical ultrasound, cervical-thoracic CT, and assay of serum Tg levels.

### Statistics analysis

SPSS ver. 26.0 statistical analysis software was used. The paired test or χ^2^ test was employed to compare groups. The Fisher test was used to compare detection efficiencies. A single-factor analysis of variance was employed to derive correlations among groups. The interobserver agreement for subjective analyses was assessed using kappa coefficients, with a k value of ≤0.20 indicating poor agreement, 0.21-0.40 fair, 0.41-0.60 moderate, 0.61-0.80 good, and ≥ 0.81 excellent agreement. *P* value of < 0.05 was considered statistically significant.

## Results

### General characteristics

Overall, 86.3% of patients had tumors of diameter ≤ 4 cm; 147 were pathologically diagnosed with lymph node metastases, 49.1% had multiple lesions, 56 evidenced thyroid capsule invasion, and 26.7% showed extra-glandular invasion. The clinical stages were I and II (67.1% and 24.2%, respectively), and There are 14 persons in phase III and IV; 86 people were classified as low risk, 22 as medium risk and 53 as high risk. A total of 105 patients were treated with ^131^I once and 56 patients several times (**Table 1**).

**Table 1:** General clinical characteristics of all included patient.

Characters			Patients (N)	(%)
Age		44.75 ± 14.43 (12-79)	161	100
Gender	male		68	42.2
	female		93	57.8
Pathological type	Papillary carcinoma		152	5.6
	Follicular carcinoma		9	94.4
TNM staging	T0		2	1.2
	T1		73	45.3
	T2		43	26.7
	T3		13	8.1
	T4		30	18.6
Clinical stages	I		108	67.1
	II		39	24.2
	III		4	2.5
	IV		10	6.2
Hazard stratification	Low risk		86	53.4
	Moderate risk		22	13.7
	High risk		53	32.9
Maximum diameter of tumor	<2		81	50.3
	2-4		58	36.0
	>4		22	13.7
Lymph node metastasis	Yes		147	91.3
	No		14	8.7
Lesion	Single		82	50.9
	Multiple		79	49.1
Tumor invading capsule	Yes		56	34.8
	No		105	65.2
Extra-glandular invasion	Yes		43	26.7
	No		118	73.3
^131^I course of treatment	1		105	65.2
	2		34	21.1
	>2		22	13.7

### Imaging of the residual thyroid, lymph nodes, and lung metastases

We compared the number of thyroid, lymph node, and lung lesions on the imaging of day 3, day 7 and day 10 by visual analysis, respectively. The results showed that imaging of the day 3 showed more residual thyroid tissue (59 vs. 57, 47 vs. 46) (P<0.001) than day 7 and day 10 , while the imaging of day 7 and day 10 showed more lymph node (36 vs. 29, 19 vs. 14) and lung (18 vs. 10, 6 vs. 4) uptake than day 3 imaging (P<0.001). The numbers of Thyroid, lymph node, and lung uptake were statistically different between imaging of day 3 and day 7 and imaging of between day 3 and day 10 (**Table 2**). The results showed that early imaging was easier to find residual thyroid tissue, and delayed imaging was easier to find lymph nodes and lung metastasis.

**Table 2:** Comparison the numbers of lesions on day 3, day 7 and day 10 images.

Time	N	Thyroid bed	Statistics	*P*	lymph nodes	Statistics	*P*	Lung	Statistics	*P*
Day 3	98	59	77.57	0.000	29	27.13	0.000	10	28.21	0.000
Day 7	98	57			36			18		
Day 3	63	47	45.67	0.000	14	9.95	0.000	4	21.25	0.000
Day 10	63	46			19			6		
Day 7	98	57	3.67	0.055	36	0.74	0.391	18	2.36	0.124
Day 10	63	46			17			6		

### Comparison of early and delayed imaging in terms of thyroid uptake grade

Through visual analysis, we can see that on the third day of imaging, there were 60 patients (60/161) with thyroid uptake grade I, 22 patients (22/161) with II grade and 79 patients (79/161) with III grade. On the 7th day, thyroid uptake was grade I in 33 patients (33/98), grade II in 28 patients (28/98) and grade III in 37 patients (37/98). In the 10th day imaging, thyroid uptake was grade I in 15 patients (15/63), II in 22 patients (22/63), and III in 26 patients (26/63). There was moderate to good interobserver agreement for all images at Whole-body images (kappa=0.60-0.80).The χ^2^ test revealed significant differences in uptake grade between 3rd and 7th day imaging and 3rd and 10th day imaging (both *P* < 0.001) (**Table 3**). Day 3 imaging revealed more grade III uptakes, but on day 7 imaging more grade I and grade II uptakes. For example, the thyroid gland of 8 patients was not shown in the imaging on the 3rd day (the uptake grade was grade I), but it was clearly shown in the imaging on the 7th day. The day 3, day 7, and day 10 images differed in terms of thyroid uptake. Day 3 imaging detected obvious residual thyroid tissue; only minimal residual tissue was apparent on day 7.

**Table 3:** Comparison of day 3, day 7 and day 10 imaging in terms of thyroid uptake grade.

	grades of thyroid gland	Day 3			χ^2^	*P*
		Grade I	Grade II	Grade III		
Day 7 (n = 98)	Grade I	30	2	1	73.630	0.000
	Grade II	7	8	13		
	Grade III	1	2	34		
Day 10 (n = 63)	Grade I	14	1	0	42.967	0.000
	Grade II	5	8	9		
	Grade III	3	1	22		

### The thyroid, lung, and liver TBRs

**Figure 1:**
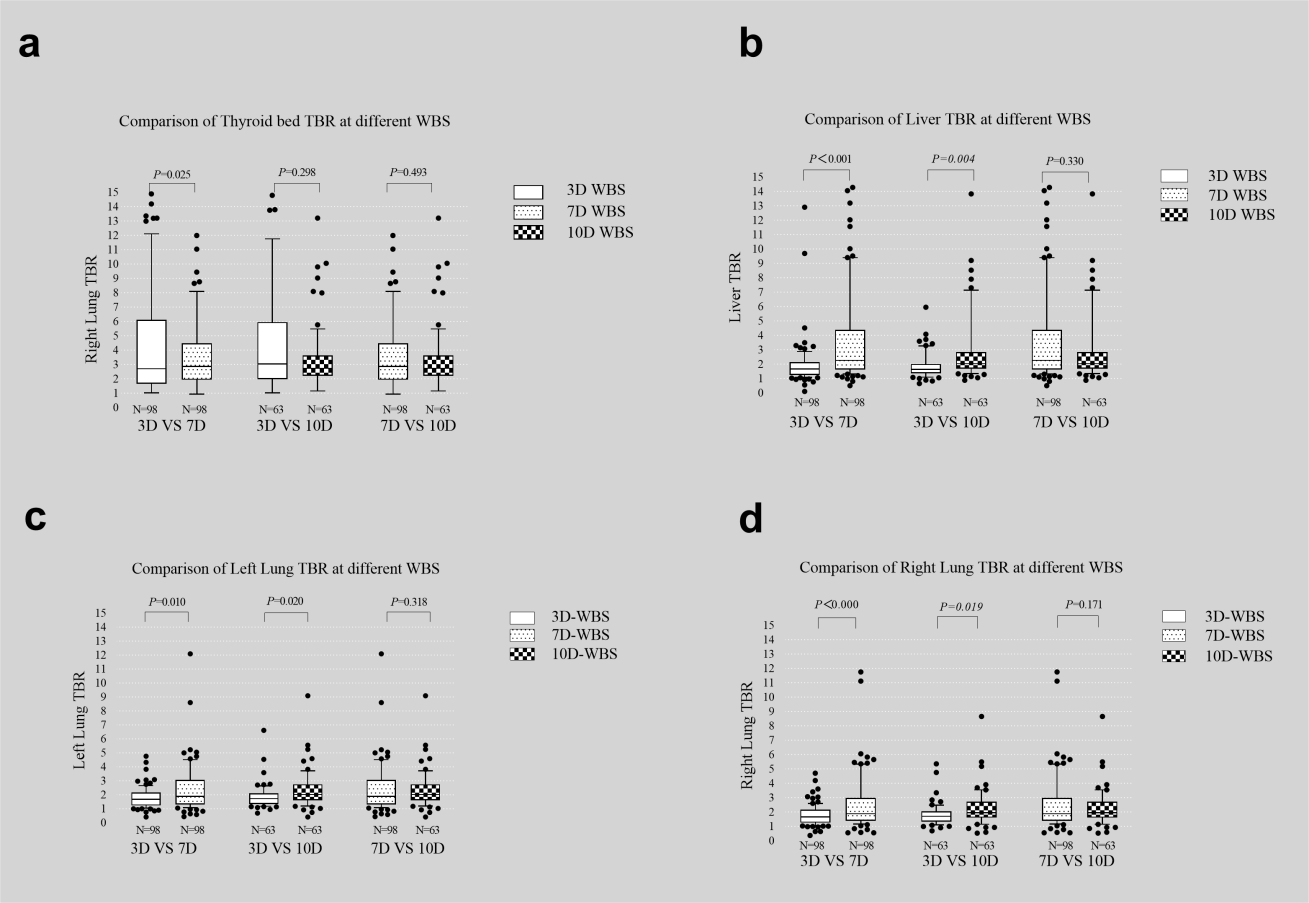
Comparison of thyroid bed **(A)**, liver **(B)**, left lung **(C)**, and right lung **(D)** TBR on day 3, day 7 and day 10 images.

Semiquantitatively, the TBR of residual thyroid tissue decreased over time. The lung TBR was highest on 7th imaging; that of the liver increased with time. The thyroid TBR differed significantly between the day 3 and day 7 images (x ± S: 7.50 ± 14.27 vs. 4.70 ± 6.54, *P* = 0.025) but not between the day 3 and day 10 or day 7 and day 10 images (x ± S: 8.99 ± 24.887 vs. 5.73 ± 12.36; 4.70 ± 6.54 vs. 5.73 ± 12.37, P = 0.298 and 0.493, respectively). The left lung, right lung and liver TBRs differed significantly between days 3 and 7 and 3 and 10 (Left lung TBR x ± S: 1.79 ± 0.73 vs. 2.85 ± 4.20, 1.89 ± 0.86 vs. 2.31 ± 1.32, *P*=0.010, 0.020; Right lung TBR x ± S: 1.77 ± 0.72 vs. 2.64 ± 2.34; 1.84 ± 0.77 vs. 2.25 ± 1.26, *P*<0.001, *P* =0.019; Liver TBR x ± S: 1.93 ± 1.53 vs. 3.79 ± 3.43, 1.85 ± 0.88 vs. 3.24 ± 0.88, *P*<0.001, *P*=0.004) but not between days 7 and 10(Left lung TBR x ± S: 2.85 ± 4.20 vs. 2.31 ± 1.32, *P*=0.318; Right lung TBR x ± S: 2.64 ± 2.34 vs. 2.25 ± 1.26, *P*=0.171; Liver TBR x ± S: 3.79 ± 3.44 vs. 3.24 ± 0.88, *P*=0.330) (**Figure 1**). A residual thyroid is most obvious early; lung metastasis is most obvious at day 7.

### Detection of metastases

Cervical ultrasound, partial lymph node biopsy, cervical-thoracic CT before ^131^I treatment, and the injection of serum irritant Tg before treatment, revealed 71 patients with cervical lymph node metastases and 41 with lung metastases. Day 3 images revealed 43 cervical lymph node, and 14 lung uptakes; the figures for days 7 and 10 were 36, and 18; and 19, and 6, respectively. The data were evaluated using the Fisher test. The sensitivities of day 3, day 7, and day 10 images in terms of metastatic lymph node detection were 29.58%, 39.02%, and 19.35%; and the specificities were 75.56%, 75.86%, and 75%, respectively. The three imaging modalities similarly detected metastatic lymph nodes (*P* = 0.465, 0.763, and 0.154, respectively). In terms of lung metastases, the sensitivities of day 3, day 7, and day 10 images were 29.58%, 38.46%, and 13.33%, and the specificities were 98.33%, 100%, and 95.83%, respectively (*P* < 0.001, < 0.001, and 0.238); day 7 imaging most effectively detected such metastases.

**Figure 2:**
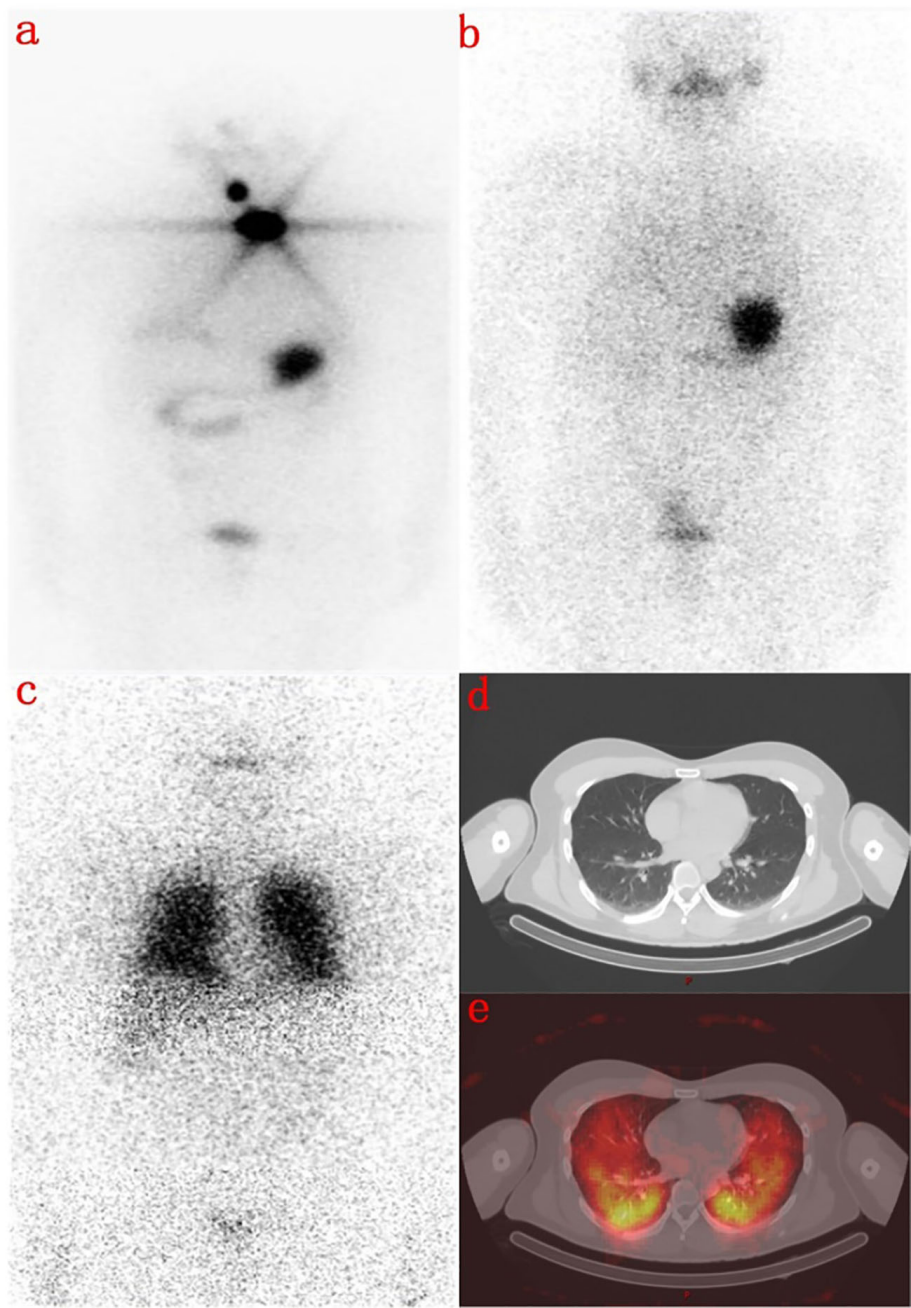
A 31-year-old male with total thyroidectomy one year ago. **(A)** shows a radioactive concentration in the residual thyroid on the first ^131^I WBS imaging performed 8 months ago. **(B)** shows that there was no abnormal concentration of radioactivity on the day 3 imaging after second ^131^I therapy. **(C)** shows that there was no display in the residual thyroid but a diffuse abnormal radioactive concentration in both lungs in the day 7 imaging. **(D)** is the Chest CT image, and **(E)** shows that lung metastasis of thyroid cancer on the day 7 with SPECT/CT fusion image.

**Figure 3:**
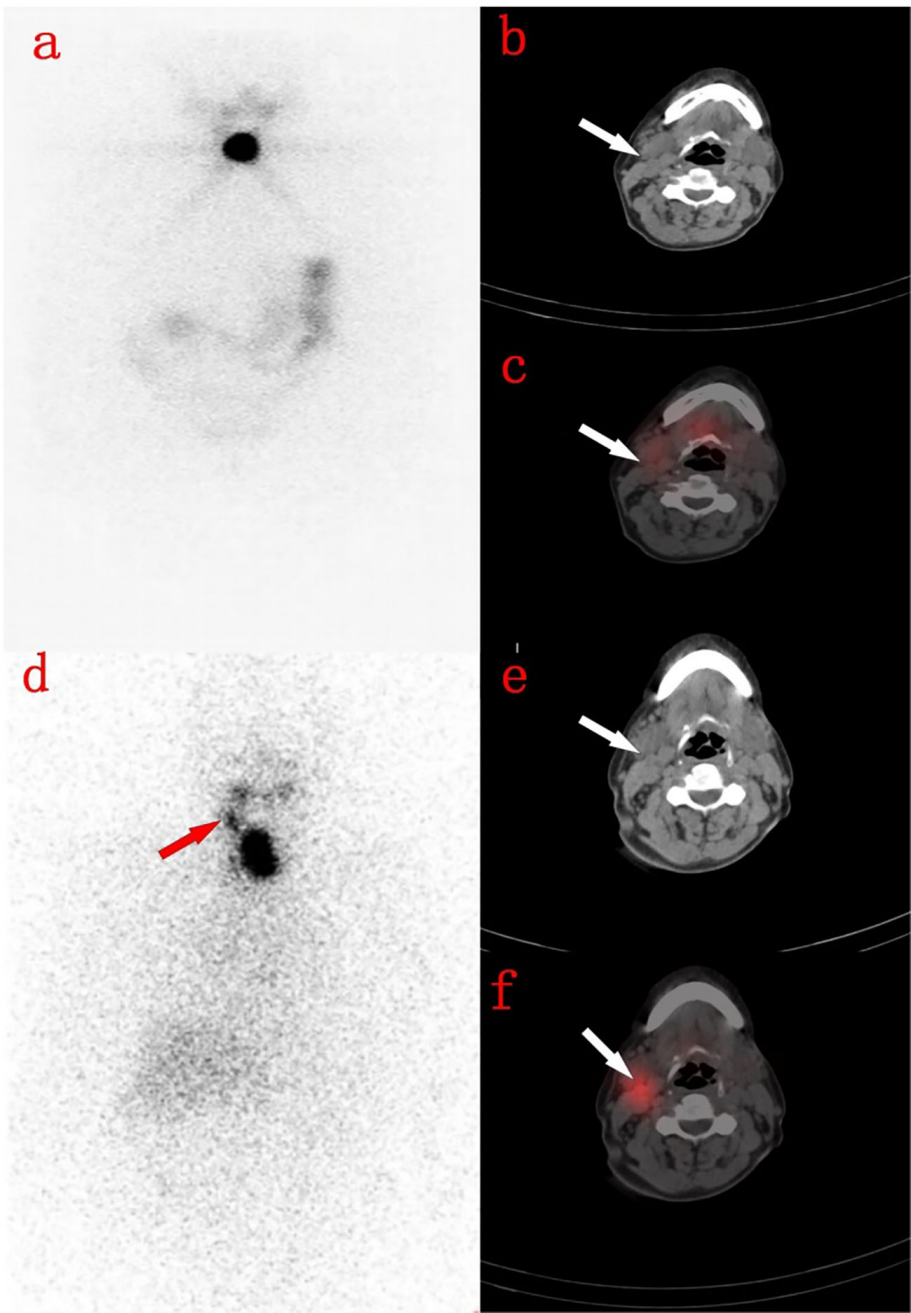
A 71-year-old woman with thyroidectomy 4 months ago. **(A)** shows the residual thyroid on the day 3 imaging. **(B)** shows the right cervical lymph nodes on the neck CT image. **(C)** shows the right cervical lymph nodes without radioactive uptake on the 3rd day SPECT/CT fusion image. **(D)** shows residual thyroid on the 7th day WBS, with radioactive accumulation in the right neck. **(E)** shows the right neck lymph nodes on the neck CT image. **(F)** shows the right neck lymph nodes showed radioactive accumulation on the 7th day SPECT/CT fusion image.

Here are two typical cases. One is a 31-year-old male with no 131 iodine uptake was found in the lungs on the 3rd day, but diffuse radioactive iodine uptake in both lungs was found on the 7th day (**Figure 2**). The other is a 71-year-old female, whose right neck was no obvious 131 iodine uptake on the 3rd day, but obvious radioactive iodine uptake in the right cervical lymph nodes on the 7th day (**Figure 3**).

## Discussion

Tx-WBS sensitively detects metastatic lesions that take up iodine and locates lesions that are not evident on cervical ultrasound, CT, or magnetic resonance imaging. Tx-WBS reveals new lesions in 6–13% of patients; 8.3% are restaged (in terms of the tumor) and follow-up treatment and management adjusted accordingly (9). Tx-WBS is used to restage DTC and plan follow-up ^131^I treatment. However, few studies have explored the optimal imaging time; a small number of retrospective studies on limited numbers of patients have shown that imaging detection efficiency varies with time.

Our larger study allowed us to draw certain conclusions. The number of residual thyroid uptakes on day 3 was twice those on days 7 and 10. In terms of lung metastasis, the sensitivity and specificity of day 7 images were significantly higher than those of day 3 and day 10 images, and the number of lymph node metastases evident on day 7 was 25–30% higher than on days 3 and 10.

Hung et al. (10) and Salvatoria et al. (11) reported that days 3–4 and 5–7 images did not differ significantly in terms of lesion numbers; day 10 images revealed fewer lesions. Lee (12) found that early imaging better detected thyroid residues and ^131^I-positive metastatic lesions than delayed imaging (the uptake rates were higher early), and that diffuse liver uptake was more common in delayed than early images (86 vs. 6%). However, Khan, Chong, and Kodani et al. (13–15) concluded that day 7 imaging was more sensitive (all tissues) than day 3 imaging. The cited authors compared only day 3 and day 7 images. We imaged on days 3, 7, and 10; early imaging revealed more residual thyroid tissue but delayed imaging more lymph node and lung lesions, unlike what was reported by Hung (10) and Salvatoria et al. (11). The cited authors performed visual analyses only. However, most cervical uptakes evident on whole-body images cannot be visually assigned to residual thyroid tissue or cervical lymph node metastases. We subjected SPECT/CT scans to visual analysis but also performed cervical and thoracic CT to evaluate the neck. This revealed that many lesions that were suspect cervical lymph nodes on whole-body scans were in fact residual thyroid lesions. Also, we derived semiquantitative lesion uptakes.

We found that the TBR of the residual thyroid bed decreased gradually with time; the lung TBR was highest on day 7, and the liver TBR increased gradually. The early high uptake by residual thyroid tissue may be followed by a gradual decrease (clearance of the initial uptake) over time from metastatic cells, reducing the TBR. The pulmonary TBR on day 7 imaging was higher than on days 3 and 10, perhaps because ^131^I uptake and excretion by pulmonary metastatic lesions are slower than those of residual tumor cells or because further acquisition of ^131^I by malignant cells increased the TBR (16). The peak ^131^I uptake (at approximately 6–7 days) decreased gradually over time; the day 10 uptake was lower than that on day 7. Some studies have suggested that on days 6–8 after ^131^I-labelled thyroid hormone is released into the blood, the physiological uptake by the liver begins to increase (17–19). It is also possible that the Na^+^/I^−^ symporter, a nonspecific iodine protein, and polyiodide peroxidase in hepatocytes absorb iodine (20), but that the iodine uptake capacity is lower than that in thyroid tissue. Thus, the liver TBR increases gradually with time.

Besides, we found that the number of lymph nodes evident on day 7 images was greater than on day 3 and day 10 images, but the sensitivity and specificity of metastatic lymph node detection did not change over time. In terms of lung metastases, the sensitivities on days 3, 7, and 10 were 29.58, 38.46, and 13.33%, respectively, and the specificities were 98.33, 100, and 95.83%. Day 7 images optimally revealed lung metastases (with statistical significance). A high TBR aids the detection of metastases; the TBR probably rises over time. The ^131^I clearance rates by physiological and pathological lesions differ, explaining the different detection rates of early and delayed scans. ^131^I absorption and excretion by lung and bone metastases are slower than those of thyroid residues; delayed scans better detect metastatic lesions. Our semiquantitative data and our comparisons of sensitivity and specificity confirmed that day 7 imaging better detected pulmonary metastases than day 3 and day 10 imaging. In summary, on visual analysis, day 3 imaging optimally detects residual thyroid tissue, but day 7 and day 10 imaging better detects cervical lymph node and lung metastases. Our semiquantitative analysis revealed that day 7 imaging better detected lung metastases than day 3 imaging.

Our work has certain limitations. Not all metastatic lesions were confirmed *via* pathological biopsy; we relied principally on SPECT/CT, cervical-thoracic CT, and laboratory indicators. Also, the ROIs were manually outlined, albeit by very experienced professionals. There is a possibility of bias.

## Conclusion

We suggest that residual thyroid tissue in patients at low risk of metastasis should be screened on day 3 only; there is no need for delayed imaging. This reduces the radiation dose and costs, and the hospital stay. For patients at high risk of metastasis, additional imaging on day 7 optimally detects metastatic lesions and thus facilitates follow-up treatment.

## Data availability statement

The raw data supporting the conclusions of this article will be made available by the authors, without undue reservation.

## Ethics statement

The studies involving human participants were reviewed and approved by The ethics committee of the First affiliated Hospital of Chongqing Medical University. Written informed consent for participation was not required for this study in accordance with the national legislation and the institutional requirements. Written informed consent was obtained from the individual(s) for the publication of any potentially identifiable images or data included in this article.

## Author contributions

ZW and HP contributed to conception and design of the study. RZ organized the database. SL and TY performed the statistical analysis. SL and RZ have been involved in drafting the manuscript and revising it critically for important intellectual content. All authors contributed to the article and approved the submitted version.

## Funding

This work is supported by Chongqing medical scientific research project (2021MSXM042)、General program of Chongqing Nature Science Foundation (cstc2020jcyj-maxmX0713, 0301020499QC-W0117).

## Conflict of interest

The authors declare that the research was conducted in the absence of any commercial or financial relationships that could be construed as a potential conflict of interest.

## Publisher’s note

All claims expressed in this article are solely those of the authors and do not necessarily represent those of their affiliated organizations, or those of the publisher, the editors and the reviewers. Any product that may be evaluated in this article, or claim that may be made by its manufacturer, is not guaranteed or endorsed by the publisher.
